# On the front line of primary health care: the profile of community health workers in rural Quechua communities in Peru

**DOI:** 10.1186/1478-4491-4-11

**Published:** 2006-05-17

**Authors:** Angela Brown, Rosa Malca, Adriana Zumaran, J Jaime Miranda

**Affiliations:** 1 Health Unlimited's Quechua Community Health Project Writing Group, Ayacucho, Perú

## Abstract

**Objective:**

To describe the profile of community health workers – health promoters, traditional birth attendants and traditional healers – in rural Quechua communities from Ayacucho, Peru.

**Methods:**

Basic quantitative and qualitative information was gathered as part of a community health project implemented between 1997 and 2002 in 40 Andean communities with information from questionnaires, personal interviews and group discussions.

**Results:**

The majority of current community health workers are men with limited education who are primarily Quechua speakers undertaking their work on a voluntary basis. Health promoters are mostly young, male, high school graduates. There exists a high drop-out rate among these workers. In contrast, traditional healers and traditional birth attendants possess an almost diametrically opposite profile in terms of age, education and drop-out rates, though males still predominate. At the community level the health promoters are the most visible community health workers.

**Conclusion:**

It is very important to consider and to be aware of the profile of community health workers in order to provide appropriate alternatives when working with these groups as well as with the indigenous population, particularly in terms of culture, language and gender issues.

## Background

Despite the abundance of research and published scientific literature concerning the development of effective health care for rural communities in developing countries, especially since the mid-1970s and the Alma Ata Conference [1,2], the literature on community health workers in Peru is limited [3].

One important approach in various countries and continents [4-6], among the different strategies attempted over the last 30 years, has been to use local human resources in the rural primary health care setting. These volunteer community health workers have been trained and given basic equipment to assist them in providing curative, preventive and health promotion activities to the community [7-10]. Traditional birth attendants have been the mainstay in the provision of rural maternal and child health care. Of all community health workers, the impact of traditional birth attendants' training is the only one that has been systematically studied and addressed [11-13].

In Peru, aside from few reports [3,14], there is no up-to-date information on the involvement and participation of community health workers in primary health care policies, projects and interventions or profiles of these actors. Yet they are directly involved in the development of those interventions at community level.

On a community health project spanning the period between 1997 and 2002, we were given the opportunity to work and interact with typical community health workers. This category includes health promoters, traditional birth attendants and traditional healers [15]. Hence, the aim of the present study was to describe the profile of community health workers in rural Quechua communities from Ayacucho, Peru.

## Methods

### The community health project

This is a descriptive study developed as part of a community health project carried out between 1997 and 2002 by Health Unlimited [15-17]. The intervention area was selected because of its history of 13 years of internal conflict since 1980 and due to its limited access to health services.

The project was developed in coordination with the local Ministry of Health department. The main strategies of the project were:

• involvement of community members in ascertaining relevant health issues so as to inform and guide the project's strategies;

• information, education and communication activities on relevant health issues such as maternal and child health, traditional medicine, food and nutrition, basic sanitation and hygiene and mental health;

• training and integration between different community health workers, including health promoters, traditional birth attendants and traditional healers;

• construction of community health posts;

• strengthening of the referral system from communities to the Ministry of Health facilities and integrating the community health workers in the process.

### Setting

Some 54.8% of the Peruvian population is poor, and 24.4% of Peru's population lives in extreme poverty. Ayacucho, the project's intervention area, has been classified as a department with generalized poverty due to its 72.5% poverty rate; 45.4% of Ayacucho's population lives in extreme poverty [18]. These figures were obtained from Peru's 2001 national survey of households, which defined poverty according to household expenditure structure and patterns and basic food needs. Households in poverty were unable to afford a basic basket of foods; those in absolute poverty were unable to afford basic foods that guaranteed an adequate intake of calories.

The project was conducted in 40 communities, covering a population of 8310. Thirty-two of the communities were part of Santillana district, a province of Huanta, and the remaining eight belonged to Tambo district in the province of La Mar, both in Ayacucho. This mountainous area, with an altitude ranging from 3200 to 4000 metres above sea level, is located in the central Peruvian Andes.

Travel time to the project area from Ayacucho city, the main contact city and capital of the department, was from three to five hours by automobile. When the project's activities were implemented, 40% (16/40) of the communities were accessible solely by foot, resulting in an additional two-and-a-half to five hours' round trip from the nearest road. There were only four basic health care facilities serving the area, and Ministry of Health resources were scarce.

### Subjects

Most of the project activities were implemented with community health workers from each community. All current and past community health workers of the 40 project communities were invited to participate in this study. For those who no longer worked as community health workers, the baseline project records were reviewed to extract basic demographic information. This information was double-checked and confirmed by a local community health worker from the same community. Hence, this paper presents information on all subjects who were performing or had performed a community health worker-related activity during the five-year project.

The local language is Quechua, an ancient Peruvian language that remains in use throughout the Andean region. All the project activities were conducted in Quechua, as this was the language common to both the project team and the local population.

### Data

The project team visited each community at least once a month. Basic quantitative and qualitative information was gathered from questionnaires, personal interviews and group discussions. Additional information was obtained from midterm and end-of-term project evaluations that used quantitative and qualitative data collection methods [16,17].

Data on sex, age and education level were collected for the three groups of community health workers. Data on specific features of their role, such as process of selection, voluntary status, number of years in their position and drop-out rate among the different workers was also gathered. For the purposes of this study, drop-outs were those community health workers no longer fulfilling the roles of that occupation. The period of activity of the community health workers was considered as the total period since they had begun working in that role, since some of them had started before the project's intervention period.

Some community health workers were involved in two or three roles concurrently – for example, as health promoters/traditional birth attendants or traditional healers/traditional birth attendants. These cases were catalogued according to the their first role, in order to avoid duplication of information.

Data were entered and analysed in the Microsoft Office Excel software package, and descriptive statistics were obtained for this report.

## Results

A total of 171 community health workers participated in the project over the five-year period. Eight people (4.7%) carried out more than a single function: two as traditional birth attendants/traditional healers/health promoters, two as traditional healers/traditional birth attendants and four as traditional birth attendants/health promoters. Table 1 presents the characteristics of community health workers by sex, age, education level, period of activity and drop-out rates.

**Table 1:** Profile of community health workers in a Quechua region, Ayacucho, Peru

	**Health promoters (n = 100)**	**Traditional birth attendants (n= 55)**	**Traditional healers (n = 16)**
Male *(%)*	84%	62%	75%
Age in years *average (mode)*	29 (25)	52 (58)	52 (57)
Age *range*	19–57	36–89	30–70
Drop-out rate (%)	24 (24%)	1 (3%)	2 (16.6%)
Years of activity *average (range)*	3.11 (1–8)	16.4 (2–42)	17.8 (4–40)
Education level *(%)*			
Illiterate	1	41.8	75
Incomplete primary school	48	52.8	25
Complete primary school	18	--	--
Incomplete high school	26	3.6	--
Complete high school	8	--	--
Higher education (university or technical school)	--	1.8	--

### Quantitative information

A notable difference in the age pattern was observed between community health workers, as well as education levels, drop-out rates and period of activity (Table 1). Health promoters were the youngest of all community health workers, with higher educational levels but also with higher drop-out rates. Men and women health promoters had similar drop-out rates (23.8% and 25%, respectively). The proportion of male health promoters was 84%; traditional healers, 75%; and traditional birth attendants, 62%.

In general, a low educational level – ranging from illiterates to incomplete high school education – was observed among the three groups of community health workers. Some 75% of traditional healers were illiterate and the remaining 25% had incomplete primary school education. Among traditional birth attendants, more than 90% fell in the illiterate and incomplete primary school education categories. In the case of health promoters, 1% were illiterate, 48% had incomplete primary school education and only 8% of these agents had completed high school.

This pattern also reflected their ability to speak other languages. Most of the health promoters were able to communicate in Quechua and Spanish, which was not necessarily the case for the other community health workers. The only agent who pursued studies further than high school level was a schoolteacher who later became a traditional birth attendant in his community.

The number of years that health promoters had worked for their community in their specific positions was variable (Table 1). Traditional birth attendants and traditional healers had the longest time in service.

Drop-out rate during the project intervention was 24% among health promoters. Traditional healers and traditional birth attendants had lower rates of drop-out, at 16.6% and 3%, respectively.

### Qualitative information

The project's activities carried out with community health agents have been described elsewhere [19-24]. The work of traditional healers and traditional birth attendants was based on a non-biomedical, non-allopathic model of the health-disease process. In contrast, health promoters tended to combine aspects of both biomedical/allopathic and indigenous cultures.

Traditional birth attendants concentrated specifically on the delivery period, and were accustomed to working with limited contact with health professionals. Women and families from the communities established contact directly with the traditional birth attendants, usually based on previous experience. This has also been described as an important determinant of skilled birth attendance in rural Cambodia [25]. Traditional birth attendants' services were required because of the cultural appropriateness of their activities, which included a vertical position of the women during labour and delivery, participation of the family and observance of certain cultural norms linked to the delivery process [19].

Traditional healers played a different role, mostly related to the understanding of health beliefs and ill-health processes in these communities. When requested, traditional healers' active participation usually consisted of providing local natural remedies.

Health promoters were more familiar with western health models and more accustomed to working towards health promotion and disease prevention, as well as dealing with basic emergencies. Some of them, based on their training and position in the community, liaised with other agents in order to provide better alternatives for people's care.

The basic requisites to become a community health worker were to be a member of the community and to speak Quechua. The selection process differed among the three types of community health workers. Traditional healers and traditional birth attendants tended to learn directly from a master in their field of work; they became community health workers later through the direct transmission of knowledge and practices. Masters have traditionally been older agents, usually relatives or close associates of the trainee. In the case of the health promoters, their positions were decided at a community assembly after a nomination and selection process.

All the community health workers carried out their work on a voluntary basis at any time of the day when they were needed. Almost all had to work at home and/or in subsistence agriculture. Health promoters were usually exempted from communal work when attending training activities. In the case of traditional community health workers, they often received gifts from their patients, usually in the form of food, such as grains or meat, or were assisted with their work on the land. These were perceived as stipends in return for their time and work, rather than as formal payments

Health promoters were the most visible health care providers in the community, due mainly to their involvement in continuous training by nongovernmental organizations or Ministry of Health teams. They were also the most frequent first point of contact for patients' referral to the Ministry of Health services and helped with certain Ministry of health programmes, such as those for nutrition and maternal health.

Traditional birth attendants and traditional healers received less attention from outsiders and preferred to provide services independently and person-to-person. In these circumstances, where the agents were mainly Quechua speakers whose services were required only for a short period, traditional healers and traditional birth attendants had little or no contact with health professionals. On the other hand, health professionals were usually not able to establish communications with these agents, due in part to language limitations but most importantly, due to the different health belief structure under which they operated.

This incomplete communication, comprehension and understanding affected interpersonal relationships as well as referral processes, especially those of high priority, such as delivery of women by a skilled professional. As part of the project implementation in the area, better communication and contact between community health workers and health professionals was sought, with positive results.

## Discussion

The majority of community health workers in the Quechua communities were men, with limited education, primarily Quechua speakers, who undertook their work voluntarily. The information and profiles presented in this paper will be useful for understanding the Quechua community health system; they provide an insight into this system that could benefit the implementation of future interventions. Knowledge about health care needs and service provision in rural settings is lacking when compared with their urban counterparts. Our findings need to be considered when designing community health interventions for this and similar areas, and in particular the language, education and sex differences should be integral to the planning of any health intervention involving the participation of community health workers.

It has been noted that community health workers and health professionals work under different models and parameters of health and disease. Sometimes it is assumed that the western or biomedical model is the best one to follow, and therefore strategies should aim to "convert" community health workers to it. This is not necessarily true, and a recent systematic review of women's position during labour showed increased benefits of the traditional vertical model when compared to the western horizontal position [25]. Hence, future intervention directed to establish or improve linkages between community health workers and health professionals should not be unilaterally directed towards the community health worker's side, but should attempt to simultaneously work with both groups.

Thus, training of community health workers needs to incorporate culturally appropriate elements as well as employ specific and simple educational techniques. Undertaking all activities in the Quechua language is crucial in order to ensure that comprehensible health promotion messages can be easily relayed to other community members, especially women. Continuous follow-up is essential to ensure that health training is applied correctly in practice and that opportunities are given to respond to problems that arise.

The higher number of males taking positions as community health workers reflects the significant gender differences within this population. These differences have been observed in other aspects of such communities, especially with regard to positions of leadership [16]. One barrier to gender equality in leadership we observed in the project was the disapproval of husbands of the participation of women in training workshops and health promoter activities. They were often critical of other males, including health personnel, being in the presence of their wives. The husbands tended to justify this viewpoint by pointing out that regular trips to receive training and participate in related activities meant their wives had to be away from their homes and leave the children without adequate support.

The differences observed between drop-out rates among community health workers needs to be explained in this particular context, paying particular attention to health promoters. As their own community selects them and they work on a voluntary basis, it is possible that without continuous training or moral support they will abandon their tasks. In contrast, traditional birth attendants and traditional healers were the most confident of the community health workers among the Quechua population, due to prevailing health beliefs that they share with their community [21,26]. This probably reflects the acceptance of their long-standing role within the indigenous health system.

The work done by the community health workers is highly valued by the population served. As has been seen in many similar programmes in the area, it is all too easy for community health workers to be seen as an extension of Ministry of Health services once they receive training, in spite of the voluntary nature of the work undertaken. A relationship based on mutual respect, aided by communicating in the local Quechua language, is a vital goal for the Ministry of Health if it is to gain the confidence of the community.

Working in these areas remains a considerable challenge, especially when taking into account the poverty, limited access to health care and geographical isolation of these indigenous people [27,28]. Cristina Torres states that "the differences in the health situation of minority groups are related to structural factors such as poverty, to factors directly attributable to the organization of heath services and their quality" [29]. All these factors mentioned are present, and moreover have been present in this indigenous society for many years.

It is the task of those designing sound public health policies to try to benefit all, especially those groups that have historically been excluded, such as the indigenous populations. By not taking account of our findings in future health policy planning or design of interventions, we feel such policies or projects will be undermined by a lack of appropriateness for these populations.

## Competing interests

The author(s) declare that they have no competing interests.

## Authors' contributions

JJ Miranda conceived the paper, organized the collection of information and wrote the initial draft of the paper in conjunction with A Brown. All authors contributed substantially to the intellectual content of the paper and the writing and finalization of the manuscript.
